# Preoperative risk stratification for pulmonary complications after neoadjuvant therapy and esophagectomy in locally advanced esophageal squamous cell carcinoma: a retrospective cohort study

**DOI:** 10.3389/fonc.2026.1911374

**Published:** 2026-09-22

**Authors:** Huan Lv, Ming Wang, Mingchuang Zhu, Tao Lu, Yong Ma, Ruirui Han

**Affiliations:** Department of Thoracic Surgery, Shanxi Province Cancer Hospital/Shanxi Hospital Affiliated to Cancer Hospital, Chinese Academy of Medical Sciences/Cancer Hospital Affiliated to Shanxi Medical University, Taiyuan, Shanxi, China

**Keywords:** esophageal squamous cell carcinoma, esophagectomy, neoadjuvant therapy, nomogram, postoperative pulmonary complications

## Abstract

**Purpose:**

Postoperative pulmonary complications (PPCs) are a major cause of morbidity among patients with esophageal squamous cell carcinoma (ESCC) undergoing esophagectomy. Risk stratification is crucial for patients receiving neoadjuvant therapy (NAT), as their perioperative course differs. We aimed to identify preoperatively available factors associated with PPCs after neoadjuvant therapy and esophagectomy and to develop a practical model for preoperative risk stratification.

**Methods:**

We retrospectively analyzed consecutive adults with locally advanced ESCC who underwent NAT [chemotherapy (NCT) or immunochemotherapy (NICT)] followed by curative-intent esophagectomy (2023–2024). PPCs were defined *a priori* and retrospectively ascertained through standardized chart review. The prediction time point was immediately before esophagectomy, and model development was restricted to preoperatively available variables. Five predictors were entered simultaneously into a multivariable logistic regression model. Performance was assessed by discrimination, calibration, and decision-curve analysis, with internal validation using 1,000 bootstrap resamples.

**Results:**

Among 232 eligible patients, 69 (29.7%) experienced PPCs. In the final model, smoking history was associated with higher odds of PPCs, whereas hypertension, NICT versus NCT, preoperative nebulization, and preoperative respiratory exercise were associated with lower odds. The model showed acceptable discrimination [apparent Area Under the Curve (AUC), 0.783; optimism-corrected AUC, 0.764] and acceptable calibration after bootstrap internal validation. Decision-curve analysis suggested potential net benefit across a range of threshold probabilities. The model was presented as a nomogram for individualized preoperative risk estimation.

**Conclusions:**

A model based on five preoperatively available variables showed acceptable performance for PPC risk stratification after neoadjuvant therapy and esophagectomy in locally advanced ESCC. The nomogram may support individualized preoperative risk estimation, but external validation and prospective impact assessment are required before clinical implementation.

## Introduction

Esophageal cancer remains a major global health burden and is among the leading causes of cancer-related mortality worldwide (1). In East Asia, esophageal squamous cell carcinoma (ESCC) predominates, and multimodal management with neoadjuvant therapy followed by esophagectomy has become standard for locally advanced disease (2, 3).

Despite advances in surgical technique and perioperative care, esophagectomy still carries substantial morbidity (4). Postoperative pulmonary complications (PPCs) are among the most frequent and clinically consequential events, often prolonging hospitalization and increasing early mortality (5). Reported PPC incidence and risk factors vary across studies, in part because definitions and adjudication thresholds differ (6). To improve comparability, consensus outcome frameworks such as the European Perioperative Clinical Outcome (EPCO) definitions have emphasized standardized PPC endpoints (7).

Existing esophagectomy-specific risk prediction tools were largely derived from heterogeneous populations and earlier treatment eras (8). Even contemporary minimally invasive series report variable predictors and outcome rates, highlighting the need for context-specific models (9, 10). Patients treated with intensified neoadjuvant regimens may have distinct perioperative physiology and care pathways, yet dedicated postoperative risk stratification data remain limited.

Previous studies have explored pulmonary risk prediction after esophagectomy. Notably, Wang et al. (11) developed a nomogram for postoperative pneumonia in patients with ESCC undergoing esophagectomy after neoadjuvant immunochemotherapy, using DLCO and the change in white blood cell count during neoadjuvant treatment as predictors. However, that model focused specifically on pneumonia and on patients receiving NICT. Whether routinely available preoperative factors can support risk stratification for a broader spectrum of PPCs across patients receiving either NCT or NICT remains less well characterized.

Accordingly, there is a practical need for a readily applicable model based on information available before surgery to identify patients at increased risk of PPCs. In this context, we conducted a retrospective cohort study of patients with locally advanced ESCC treated with neoadjuvant therapy followed by esophagectomy to identify preoperatively available factors associated with PPCs and to develop a model for preoperative risk stratification.

## Materials and methods

### Study design and setting

This retrospective observational study included consecutive patients treated at the Department of Thoracic Surgery, Shanxi Province Cancer Hospital between January 2023 and December 2024.

### Eligibility criteria

Patients were eligible if they: (1) had clinically diagnosed resectable locally advanced ESCC (AJCC stage IIb–IIIc); (2) received neoadjuvant therapy followed by curative-intent esophagectomy with systematic lymphadenectomy; and (3) were aged ≥18 years. Exclusion criteria included advanced or metastatic disease, early-stage upfront surgery, severe cardiopulmonary insufficiency or severe arrhythmia precluding surgery, refusal of surgery after neoadjuvant therapy, incomplete key data, prior malignancy, or severe psychiatric disease.

### Neoadjuvant therapy and surgery

Neoadjuvant therapy consisted of chemotherapy alone (neoadjuvant chemotherapy, NCT) or chemotherapy combined with immunotherapy (neoadjuvant immunochemotherapy, NICT), selected according to multidisciplinary evaluation and contemporary practice. All patients subsequently underwent curative-intent esophagectomy (McKeown or Ivor Lewis) with systematic lymphadenectomy.

### Perioperative respiratory management

Two preoperative pulmonary optimization measures were abstracted from perioperative nursing/physiotherapy records. Preoperative respiratory exercise was defined as supervised breathing training (with or without inspiratory muscle training devices) performed preoperatively. Preoperative nebulization was defined as receipt of any nebulized therapy preoperatively as part of airway humidification or secretion management. Because these measures were implemented as routine care-process measures rather than assigned according to a randomized or standardized study protocol, associations involving these variables should not be interpreted as causal treatment effects and may be subject to confounding by indication, healthy-user bias, and differences in adherence.

### Outcome definition

The primary outcome was PPCs occurring from the end of surgery until hospital discharge. PPCs were defined *a priori* for the present study as the occurrence of any of the following events, aligned with commonly used perioperative/esophagectomy consensus definitions: (i) pneumonia (new/progressive pulmonary infiltrate on chest radiograph or CT plus ≥2 of fever >38 °C, leukocytosis/leukopenia, purulent sputum, positive respiratory culture, or initiation/escalation of antibiotic therapy); (ii) atelectasis (radiographic evidence judged clinically relevant and prompting therapeutic bronchoscopy and/or escalation of respiratory physiotherapy beyond routine care); (iii) acute respiratory distress syndrome (ARDS, Berlin definition); or (iv) postoperative respiratory failure (unplanned reintubation, tracheostomy, or invasive mechanical ventilation >48 hours after surgery). Events were ascertained by standardized chart review of progress notes, imaging reports, microbiology results, and respiratory support records and adjudicated by two investigators, with disagreements resolved by consensus.

### Covariates

Baseline covariates included age, sex, body mass index (BMI), smoking history, alcohol use, and comorbidities (diabetes, hypertension, and cardiovascular disease), as well as clinical staging. Perioperative and pathologic variables, including surgical approach, operative time, pathologic response, ypT/ypN stage, tumor differentiation, vascular invasion, and perineural invasion (PNI), were recorded for descriptive purposes but were not considered candidate predictors for model development.

### Statistical analysis

The prediction time point was defined as the preoperative period immediately before esophagectomy. Accordingly, only variables available by this time point were eligible for model development, and postoperative pathological variables were excluded. Continuous variables are presented as mean ± standard deviation or median (interquartile range), as appropriate, and categorical variables as number (percentage). Between-group comparisons were performed using the Student’s t test or Mann–Whitney U test for continuous variables and the chi-square test or Fisher’s exact test for categorical variables, as appropriate.

Preoperatively available variables were first evaluated using univariable logistic regression. Variables associated with PPCs at P<0.05 in the univariable analyses were carried forward to multivariable modeling. To limit model complexity and reduce the risk of overfitting given that 69 of the 232 patients developed PPCs, the resulting five predictors were entered simultaneously into the multivariable logistic regression model without further stepwise selection.

Because smoking-stratified patterns suggested possible differences in the associations of the two respiratory care-process variables with PPCs, smoking × preoperative respiratory exercise and smoking × preoperative nebulization interaction terms were evaluated separately as exploratory analyses. Neither interaction term was included in the primary prediction model. Model-based adjusted probabilities derived from these exploratory analyses were interpreted descriptively as adjusted associations rather than estimates of causal intervention effects.

Model discrimination was quantified using the area under the receiver operating characteristic curve (AUC), and calibration was assessed using calibration plots and the calibration slope. Internal validation was performed using 1,000 bootstrap resamples to quantify optimism and obtain optimism-corrected estimates of model performance. Potential decision-analytic usefulness was assessed using decision-curve analysis (DCA), which estimates net benefit across a range of threshold probabilities relative to default strategies, and a clinical impact curve, which illustrates the number of patients classified as high risk and the corresponding number of true PPC cases at different thresholds. These analyses were interpreted as measures of potential decision support rather than evidence that model-guided management reduces PPC incidence. All statistical tests were two-sided, with P<0.05 considered statistically significant. Analyses were performed using R (R Foundation for Statistical Computing, Vienna, Austria).

## Results

### Study population

Among 635 esophageal cancer cases screened during the study period, 232 patients met inclusion criteria (**Figure 1**). Overall, 69 patients (29.7%) experienced PPCs. The median time to first PPC was postoperative day (POD) 4 (IQR, 2–7). Among these patients, 41 (59.4%) first occurred on POD 1–4, 13 (18.8%) on POD 5–7, and 15 (21.7%) after POD 7.

**Figure 1:**
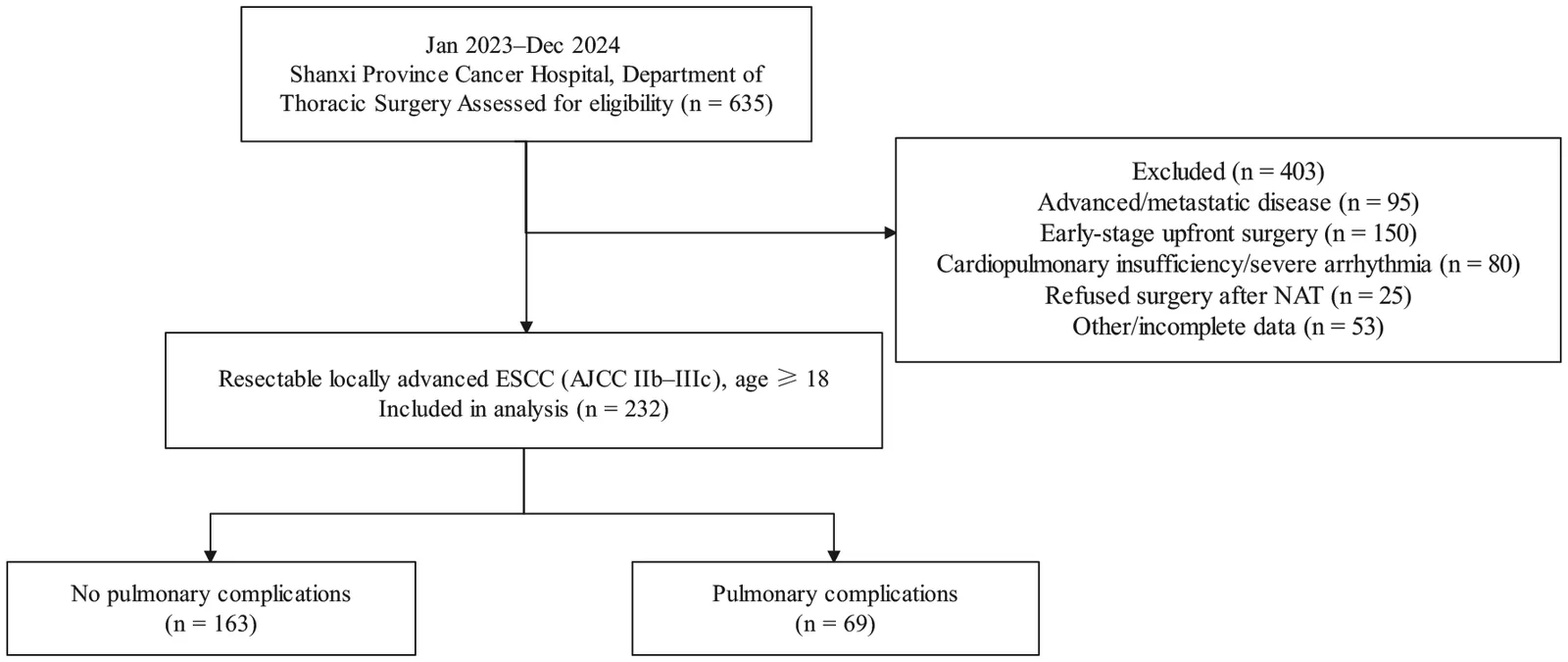
Study flowchart of patient selection.

### Baseline and perioperative characteristics

Baseline clinical characteristics are summarized in **Table 1** and perioperative/pathologic characteristics in **Table 2**. Patients who developed PPCs were more likely to have a history of smoking and were less likely to have received preoperative nebulization or respiratory exercise training.

**Table 1:** Baseline characteristics of the study cohort by PPC status.

Variables	Total (n = 232)	No (n = 163)	Yes (n = 69)	P
Sex, n(%)				0.145
Male	159 (68.53)	107 (65.64)	52 (75.36)	
Female	73 (31.47)	56 (34.36)	17 (24.64)	
Age, n(%)				0.324
≤65	140 (60.34)	95 (58.28)	45 (65.22)	
>65	92 (39.66)	68 (41.72)	24 (34.78)	
Smoking history, n(%)				0.031
No	99 (42.67)	77 (47.24)	22 (31.88)	
Yes	133 (57.33)	86 (52.76)	47 (68.12)	
Alcohol drinking, n(%)				0.403
No	144 (62.07)	104 (63.80)	40 (57.97)	
Yes	88 (37.93)	59 (36.20)	29 (42.03)	
BMI, n(%)				0.297
≤24	143 (61.64)	104 (63.80)	39 (56.52)	
>24	89 (38.36)	59 (36.20)	30 (43.48)	
Diabetes, n(%)				0.319
No	212 (91.38)	147 (90.18)	65 (94.20)	
Yes	20 (8.62)	16 (9.82)	4 (5.80)	
Hypertension, n(%)				0.010
No	180 (77.59)	119 (73.01)	61 (88.41)	
Yes	52 (22.41)	44 (26.99)	8 (11.59)	
Tumor location, n(%)				0.455
Upper	23 (9.91)	17 (10.43)	6 (8.70)	
Middle	151 (65.09)	109 (66.87)	42 (60.87)	
Lower	58 (25.00)	37 (22.70)	21 (30.43)	
Tumor length, n(%)				0.147
≤3cm	91 (39.22)	59 (36.20)	32 (46.38)	
>3cm	141 (60.78)	104 (63.80)	37 (53.62)	
Neoadjuvant therapy, n(%)				<.001
NCT	100 (43.10)	58 (35.58)	42 (60.87)	
NICT	132 (56.90)	105 (64.42)	27 (39.13)	
Preoperative nebulization, n(%)				<.001
No	130 (56.03)	79 (48.47)	51 (73.91)	
Yes	102 (43.97)	84 (51.53)	18 (26.09)	
Preoperative respiratory exercise, n(%)				0.018
No	97 (41.81)	60 (36.81)	37 (53.62)	
Yes	135 (58.19)	103 (63.19)	32 (46.38)	

**Table 2:** Perioperative and pathologic characteristics of the study cohort by PPC status.

Variables	Total (n = 232)	No (n = 163)	Yes (n = 69)	P
Surgical methods, n(%)				0.710
Ivor Lewis	10 (4.31)	6 (3.68)	4 (5.80)	
McKeown	222 (95.69)	157 (96.32)	65 (94.20)	
Arrhythmia, n(%)				1.000
No	224 (96.55)	157 (96.32)	67 (97.10)	
Yes	8 (3.45)	6 (3.68)	2 (2.90)	
Anastomotic fistula, n(%)				0.969
No	192 (82.76)	135 (82.82)	57 (82.61)	
Yes	40 (17.24)	28 (17.18)	12 (17.39)	
LN resection, n(%)				0.247
>24	91 (39.22)	60 (36.81)	31 (44.93)	
≤24	141 (60.78)	103 (63.19)	38 (55.07)	
Vascular invasion, n(%)				0.026
No	205 (88.36)	149 (91.41)	56 (81.16)	
Yes	27 (11.64)	14 (8.59)	13 (18.84)	
Perineural invasion, n(%)				0.004
No	206 (88.79)	151 (92.64)	55 (79.71)	
Yes	26 (11.21)	12 (7.36)	14 (20.29)	
pCR, n(%)				0.058
No	176 (75.86)	118 (72.39)	58 (84.06)	
Yes	56 (24.14)	45 (27.61)	11 (15.94)	
MPR, n(%)				0.103
No	129 (55.60)	85 (52.15)	44 (63.77)	
Yes	103 (44.40)	78 (47.85)	25 (36.23)	
ypT stage, n(%)				0.088
T0	43 (18.53)	33 (20.25)	10 (14.49)	
T1	53 (22.84)	38 (23.31)	15 (21.74)	
T2	53 (22.84)	37 (22.70)	16 (23.19)	
T3	80 (34.48)	55 (33.74)	25 (36.23)	
T4	3 (1.29)	0 (0.00)	3 (4.35)	
ypN stage, n(%)				0.248
N0	144 (62.07)	103 (63.19)	41 (59.42)	
N1	56 (24.14)	42 (25.77)	14 (20.29)	
N2	28 (12.07)	16 (9.82)	12 (17.39)	
N3	4 (1.72)	2 (1.23)	2 (2.90)	

### Risk factors for PPCs

Five preoperatively available variables were associated with PPCs in the univariable analyses and were carried forward to multivariable modeling: smoking history, hypertension, neoadjuvant therapy regimen, preoperative nebulization, and preoperative respiratory exercise. In the final multivariable model, smoking history was associated with higher odds of PPCs, whereas hypertension, NICT versus NCT, preoperative nebulization, and preoperative respiratory exercise were associated with lower odds of PPCs (**Figure 2**).

**Figure 2:**
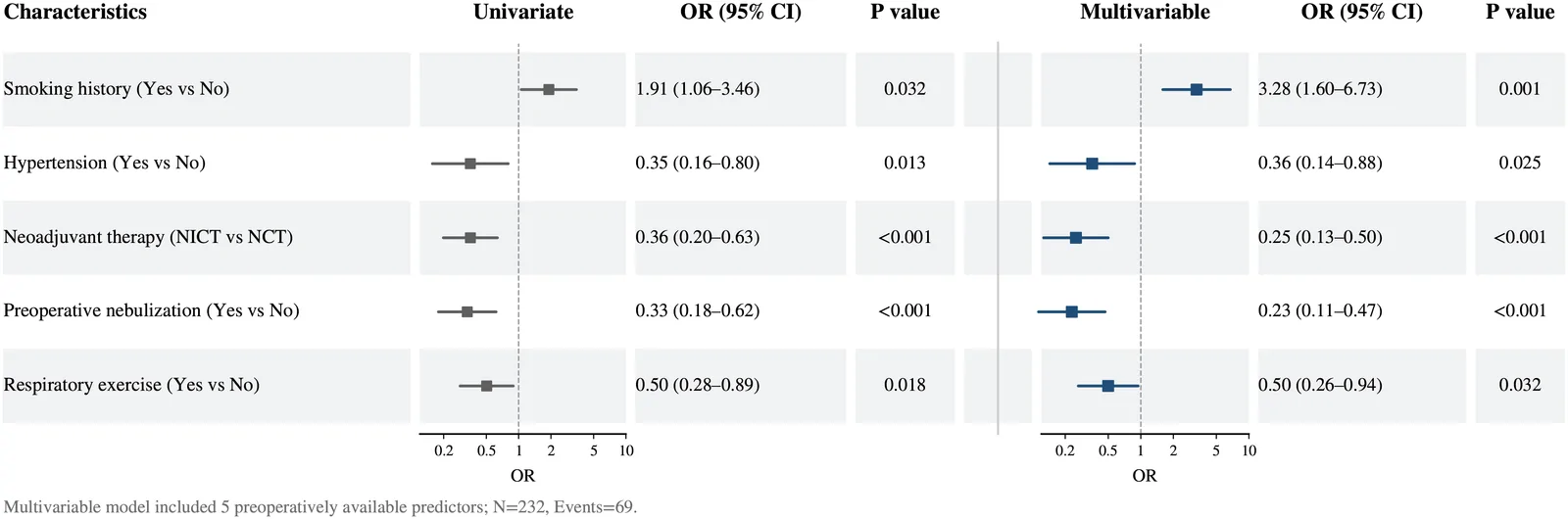
Forest plot showing univariable and multivariable logistic regression analyses for PPCs.

### Nomogram development and model performance

The preoperative prediction model included five predictors: smoking history, hypertension, neoadjuvant treatment regimen, preoperative nebulization, and preoperative respiratory exercise. The final model was visualized as a nomogram (**Figure 3**). The apparent AUC was 0.783 (95% CI, 0.716–0.848; **Figure 4**). Bootstrap internal validation yielded an optimism-corrected AUC of 0.764 and an optimism-corrected calibration slope of 0.907, suggesting modest model optimism (**Supplementary Figure S1**). Calibration analyses showed acceptable agreement between predicted and observed risks, and decision-curve analyses suggested potential net benefit across a range of threshold probabilities (**Supplementary Figures S1, S2**).

**Figure 3:**
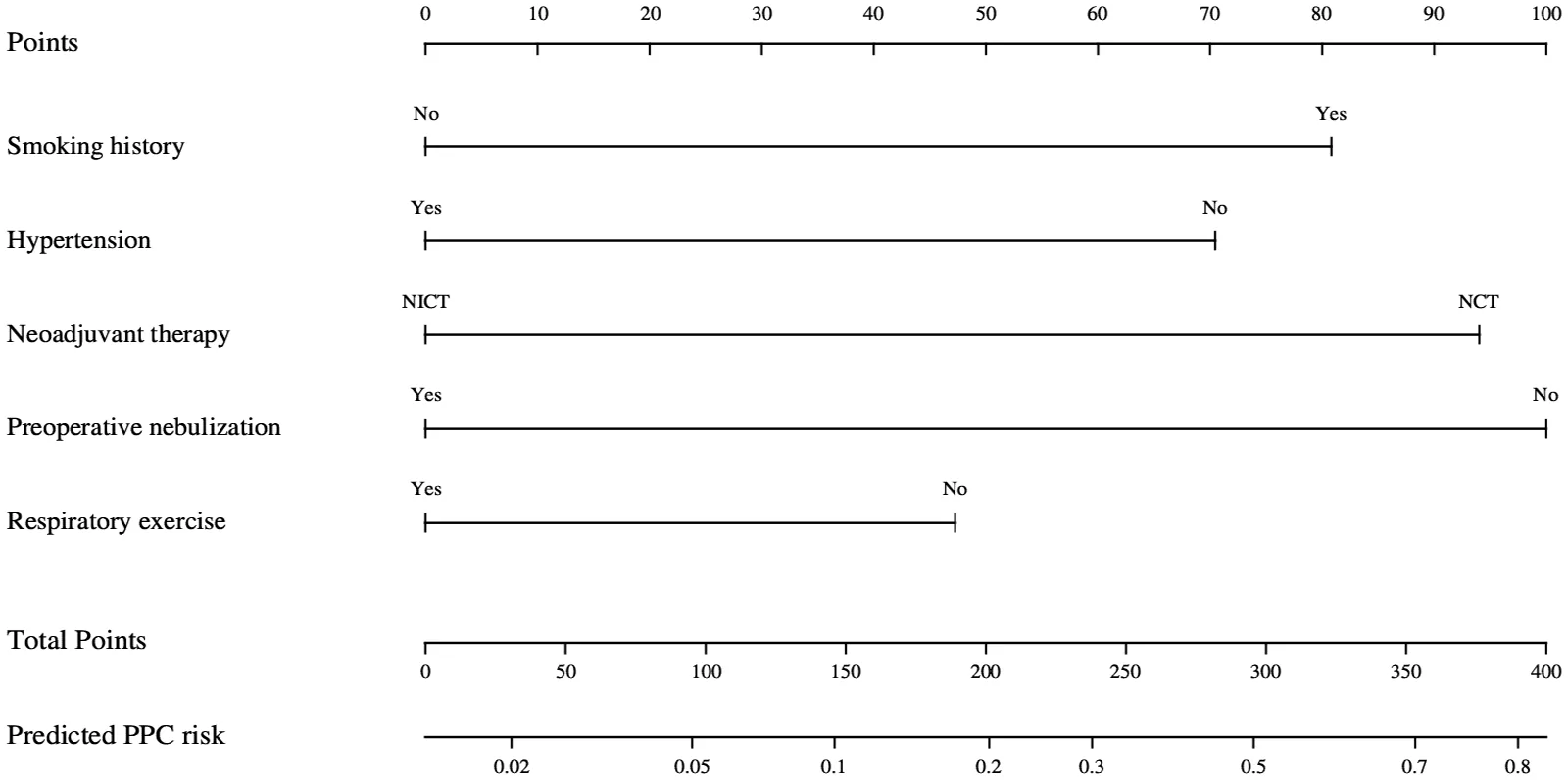
Nomogram for individualized preoperative prediction of PPC risk.

**Figure 4:**
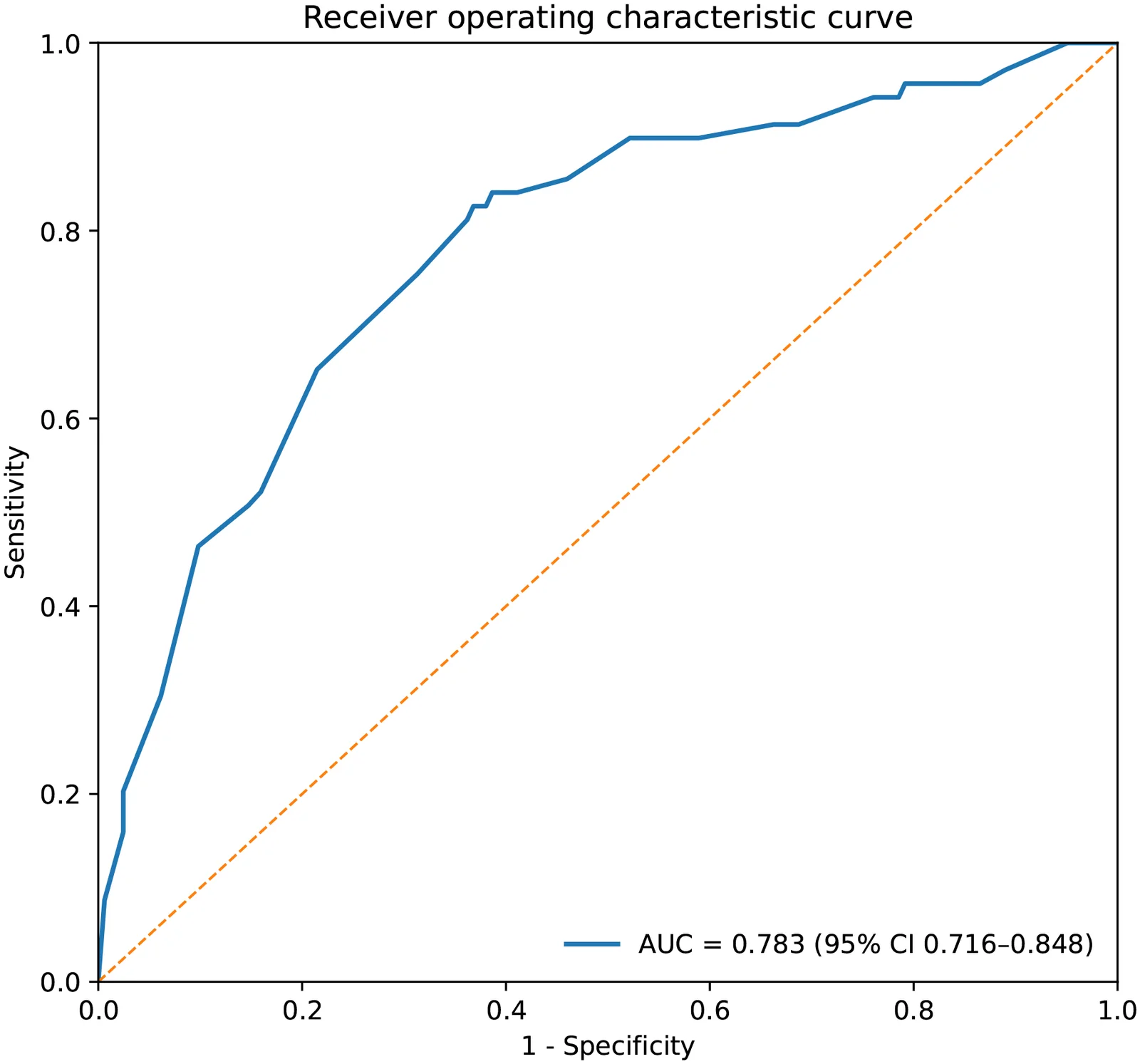
Receiver operating characteristic (ROC) curve for the final PPC prediction model.

In separate exploratory analyses, the smoking × preoperative respiratory exercise interaction was not statistically significant (OR, 0.46; 95% CI, 0.12–1.74; P = 0.255). The smoking × preoperative nebulization interaction was also not statistically significant (P = 0.772). Accordingly, the smoking-stratified probability estimates should be interpreted as descriptive exploratory findings rather than evidence of effect modification (**Figure 5**).

**Figure 5:**
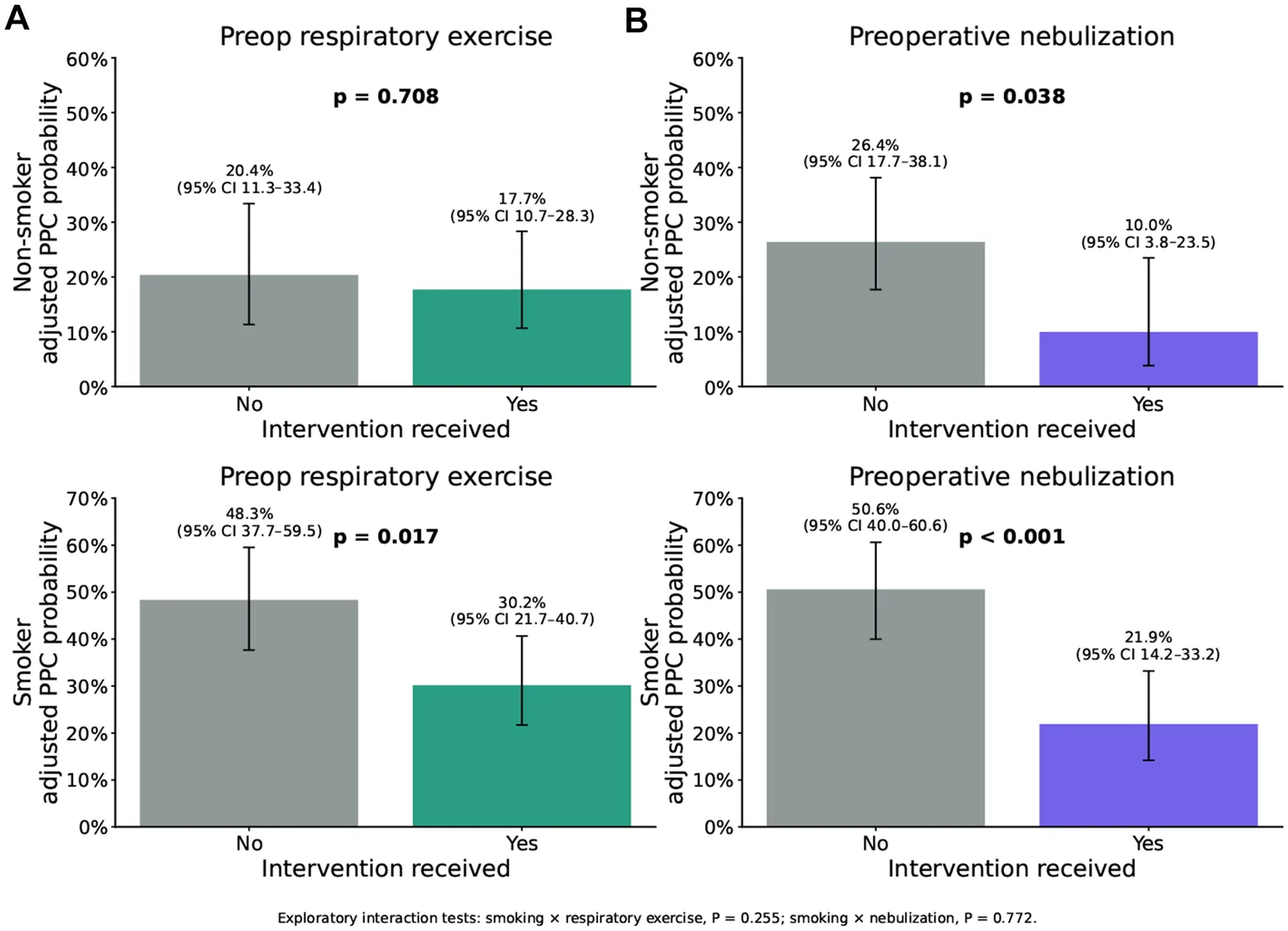
Marginal-standardized adjusted probabilities of PPCs according to smoking status and preoperative respiratory exercise **(A)** or preoperative nebulization **(B)**.

## Discussion

An important methodological consideration was the alignment of predictor availability with the prediction time point. The median time to first PPC was POD 4, and 78.3% of patients who developed PPCs had experienced their first event by the end of POD 7. Because postoperative pathological information typically becomes available only after many PPCs have already occurred, model development was deliberately restricted to variables available before esophagectomy. Accordingly, the model should be interpreted as a preoperative risk-stratification tool rather than an early postoperative reassessment model.

The present study should also be considered in the context of previous prediction work. Wang et al. (11) developed a nomogram for postoperative pneumonia in 78 patients with ESCC treated with neoadjuvant immunochemotherapy, with DLCO and the change in white blood cell count during neoadjuvant treatment retained as predictors. Their study established the feasibility of individualized pulmonary risk prediction in this specific treatment setting. The present study addresses a related but distinct clinical question. We included patients receiving either NCT or NICT and evaluated a broader composite PPC endpoint encompassing pneumonia, clinically relevant atelectasis, ARDS, and postoperative respiratory failure. In addition, the model was deliberately restricted to variables available before esophagectomy to ensure temporal alignment between predictor availability and outcome occurrence. Thus, rather than replacing or demonstrating superiority over the pneumonia-specific model of Wang et al., our model complements previous work by extending preoperative risk stratification to a broader pulmonary outcome and a broader neoadjuvant-treated ESCC population.

Smoking exposure was a consistent risk marker for PPCs, aligning with prior thoracic surgical series and perioperative evidence (12). Smoking-related airway inflammation, impaired mucociliary clearance, and reduced pulmonary reserve plausibly increase susceptibility to pneumonia, atelectasis, and respiratory failure in the setting of postoperative pain, transient hypoventilation, and fluid shifts. Clinically, these findings reinforce the value of systematic smoking history capture and, when feasible, a structured cessation pathway within prehabilitation (13).

From a pathway perspective, perioperative pulmonary optimization and prehabilitation strategies have long been investigated as approaches to mitigate pulmonary morbidity after esophagectomy (14, 15). Exercise- and nutrition-based prehabilitation has also been evaluated in a randomized trial of patients undergoing esophagogastric cancer surgery, supporting the broader rationale for preoperative functional optimization (16). Beyond short-term complications, PPCs have also been associated with worse long-term survival after esophagectomy in observational studies and meta-analyses, underscoring the clinical importance of early detection and mitigation (17).

In addition, the neoadjuvant regimen was associated with PPC risk in our cohort, with NICT showing lower odds of PPCs than conventional NCT. This association should not be interpreted as evidence of a causal protective effect of NICT, because differences in patient selection, treatment tolerance, perioperative management, and other residual confounding may have contributed to the observed association. Previous systematic reviews and meta-analyses have supported the feasibility and acceptable perioperative safety of neoadjuvant immunochemotherapy or immunotherapy in resectable ESCC (18, 19), and prospective randomized evidence has further strengthened the evidence base for this treatment strategy (20). However, these studies do not establish that NICT itself reduces the risk of PPCs. Therefore, the association observed in the present cohort should be interpreted cautiously and requires confirmation in larger multicenter studies.

Two preoperative care-process measures, respiratory exercise and nebulization, were associated with lower odds of PPCs in the present cohort. These findings should not be interpreted as evidence of causal efficacy. Because these measures were neither randomized nor assigned according to a standardized protocol, patients who received or adhered to them may have differed systematically from those who did not. In particular, healthier patients, those with better functional reserve, or those more willing and able to participate in preoperative training may have been overrepresented among patients completing respiratory exercise, giving rise to healthy-user and adherence bias (21). Conversely, nebulization may have been preferentially prescribed to patients with respiratory symptoms or secretion burden, introducing confounding by indication. Although the multivariable model accounted for the other predictors included in the model, unmeasured factors such as frailty, baseline pulmonary function, functional status, and adherence intensity remained unavailable, and residual confounding cannot be excluded. Accordingly, these variables should be regarded as care-process predictors within the present risk model rather than modifiable factors with established therapeutic benefit. Prospective studies with standardized intervention protocols are required to determine whether either measure causally reduces PPCs.

Although smoking-stratified estimates differed numerically, neither exploratory interaction provided statistically significant evidence of effect modification, and the smoking × respiratory exercise interaction was estimated imprecisely. These subgroup patterns should therefore be regarded as exploratory rather than as evidence of differential effects according to smoking status.

The decision-curve and clinical impact analyses should be interpreted as measures of potential decision support rather than evidence of clinical effectiveness. DCA estimates the net benefit of model-based risk classification across specified threshold probabilities by balancing true-positive and false-positive classifications relative to default strategies. Similarly, the clinical impact curve illustrates how many patients would be classified as high risk and how many of these would actually experience PPCs. Neither analysis demonstrates that modifying perioperative management on the basis of the model reduces PPC incidence. Accordingly, the present model should be considered a tool for preoperative risk stratification and hypothesis generation. Prospective impact studies are required to determine whether model-guided assessment or management improves patient outcomes.

## Limitations

Several limitations deserve emphasis. First, the retrospective, single-center design limits generalizability and permits residual confounding, selection bias, and potential outcome misclassification despite standardized PPC ascertainment and adjudication. Multicenter cohorts using harmonized PPC definitions are needed to assess transportability. Second, preoperative respiratory exercise and nebulization were routine care-process measures rather than randomized interventions. Their associations with PPCs may therefore be affected by healthy-user bias, adherence bias, confounding by indication, and unmeasured differences in pulmonary function, frailty, or functional status. The present prediction model was not designed to estimate causal treatment effects. Third, model development was based on 69 patients who developed PPCs, which constrained model complexity and may still permit some overfitting despite bootstrap internal validation. The modest number of outcome events also limited the precision of interaction estimates, as reflected by the wide confidence interval for the smoking × respiratory exercise interaction. Accordingly, subgroup and interaction analyses should be considered exploratory. Finally, although decision-curve and clinical impact analyses suggested potential decision-analytic usefulness, the study did not evaluate whether model-guided changes in perioperative management improve clinical outcomes. External validation and prospective impact assessment in independent cohorts are therefore required before clinical implementation.

## Conclusions

Among patients with locally advanced ESCC undergoing esophagectomy after neoadjuvant therapy, a model based on five preoperatively available variables provided acceptable discrimination and calibration for PPC risk stratification. The model may support preoperative identification of patients at higher risk of PPCs, although external validation and prospective impact assessment are required before clinical implementation.

## Data Availability

The raw data supporting the conclusions of this article will be made available by the authors, without undue reservation.
